# Using Geographic Information Systems for Exposure Assessment in Environmental Epidemiology Studies

**DOI:** 10.1289/ehp.6738

**Published:** 2004-04-15

**Authors:** John R. Nuckols, Mary H. Ward, Lars Jarup

**Affiliations:** ^1^Department of Environmental and Radiological Health Sciences, Colorado State University, Fort Collins, Colorado, USA; ^2^Occupational and Environmental Epidemiology Branch, Division of Cancer Epidemiology and Genetics, National Cancer Institute, National Institutes of Health, Department of Health and Human Services, Bethesda, Maryland, USA; ^3^Small Area Health Statistics Unit, Department of Epidemiology and Public Health, Imperial College London, London, United Kingdom

**Keywords:** environmental epidemiology, exposure assessment, geographic information systems

## Abstract

Geographic information systems (GIS) are being used with increasing frequency in environmental epidemiology studies. Reported applications include locating the study population by geocoding addresses (assigning mapping coordinates), using proximity analysis of contaminant source as a surrogate for exposure, and integrating environmental monitoring data into the analysis of the health outcomes. Although most of these studies have been ecologic in design, some have used GIS in estimating environmental levels of a contaminant at the individual level and to design exposure metrics for use in epidemiologic studies. In this article we discuss fundamentals of three scientific disciplines instrumental to using GIS in exposure assessment for epidemiologic studies: geospatial science, environmental science, and epidemiology. We also explore how a GIS can be used to accomplish several steps in the exposure assessment process. These steps include defining the study population, identifying source and potential routes of exposure, estimating environmental levels of target contaminants, and estimating personal exposures. We present and discuss examples for the first three steps. We discuss potential use of GIS and global positioning systems (GPS) in the last step. On the basis of our findings, we conclude that the use of GIS in exposure assessment for environmental epidemiology studies is not only feasible but can enhance the understanding of the association between contaminants in our environment and disease.

Environmental epidemiology is an area of epidemiology concerned with the study of associations between environmental exposures and health outcomes, with the purpose of further understanding the etiology of disease. The term “environment” implies a spatial context. Thus, the study of interactions between humans and their environment requires spatial information and analysis. Geographic information system (GIS) software allows environmental and epidemiologic data to be stored, analyzed, and displayed spatially. The logical structure and functionality of a GIS are shown in Figure 1 (Falbo et al. 1991). Data collection can be accomplished by importing tabular or digital data that are referenced with map coordinates defining their geographic position. The data are entered into a database where they are stored as a map with a specified theme (termed “data layer”). Tabular (attribute) data corresponding to the theme can be stored with each data layer. Analytical functions within the software can be used to process and manipulate both map and attribute data through linkages established within the GIS. Two types of output are common: tabular (summary data, statistics, reports) and cartographic (maps, map files, and map overlays). Several publications describe the structure and functionality of a GIS more thoroughly (Chrisman 2002; DeMers 2000). Vine et al. (1997) provide an overview of the use of specific functions in GIS software that could be useful in environmental epidemiologic research. Beyea and Hatch (1999) provide an in-depth discussion of geographic modeling for exposure assessment in environmental epidemiology, as well as an extensive literature review. Briggs and Elliot (1995) provide a review of spatial analysis and mapping in environmental health.

GIS have been used at different levels of sophistication in environmental epidemiology studies. These uses range from simply locating the study population by geocoding addresses (assigning mapping coordinates) to using proximity to contaminant source as a surrogate for exposure (Bell et al. 2001; Comba et al. 2003; Langholz et al. 2002; Xiang et al. 2000) to integrating environmental monitoring data into the analysis of the health outcomes (Floret et al. 2003; Gallagher et al. 1998; Reynolds et al. 2002a, 2002b, 2003). However, most of the latter studies have been ecologic in design; relatively few studies have used GIS in estimating environmental levels of a contaminant at the individual level (Nyberg et al. 2000; Reif et al. 2003; Rogers et al. 2000). A number of studies have used GIS to design exposure metrics for use in epidemiologic studies (Bellander et al. 2001; Brody et al. 2002; Cicero-Fernandez et al. 2001; Gunier et al. 2001; Inserra et al. 2002; Kohli et al. 1997; Rull and Ritz 2003; Swartz et al. 2003; Ward et al. 2000). Although yet to be applied in the context of an epidemiologic analysis, several studies have investigated the use of GIS in estimating activity patterns of the study population for potential linkage to environmental data to refine personal exposure estimates (Elgethun et al. 2003; Phillips et al. 2001). Similarly, the use of GIS in spatial statistics for linking exposure and health data in the context of epidemiologic analysis is a growing field of research (Ali et al. 2002; Christakos and Serre 2000; Elliott et al. 2001). This article is a discussion of the fundamentals of the scientific disciplines required to use GIS in exposure assessment for epidemiologic studies and explores how a GIS can be used to accomplish several steps in the exposure assessment process (those shaded blue in Figure 2). Specifically these steps are *a*) defining the study population, *b*) identifying source and potential routes of exposure, *c*) estimating environmental levels of target contaminants, and *d* ) estimating personal exposures.

## Fundamentals of GIS Application in Exposure Assessment

Using GIS in exposure assessment for epidemiologic studies requires knowledge and expertise in at least three core scientific areas: geospatial sciences, environmental sciences, and epidemiology.

### Geospatial Science

For a GIS to accurately represent occurrences on the earth’s surface, the location of data must be reliable, accurate, and pertinent (Falbo et al. 1991). Geospatial science is the systematic study of geographic variables relating to, occupying, or having the character of space. Fundamental elements of geospatial sciences relevant to GIS applications in exposure assessment include data representation, scale, and accuracy. Data representation is the format of the unit of analysis used in the GIS. The most commonly used representations of space in a GIS are the raster and vector data models. In the raster model, grid cells serve as the basic units of analysis. An example would be pixels of remotely sensed imagery from satellite imagery. The vector model uses points, lines, or polygons based on continuous geometry of space to represent data. Other, more specialized data models are available in most GIS software. For example, the triangulated irregular network (TIN) model provides an efficient means of representing elevation data often used for terrain analysis. GIS software contain algorithms for translating between formats, for example, raster → vector, vector → raster, point → TIN, although some error may be introduced by these data transformation processes. More complete information on data models can be found in textbooks such as those by Chrisman (2002) and DeMers (2000).

Selection of scale is perhaps the most important factor in creating and analyzing GIS databases for exposure assessment and epidemiology. The following is a list of definitions of the the scaling factors most likely to be encountered in an epidemiology study:

- Cartographic scale: Traditional map scale ratio relates the size of a feature on the ground to the size of a feature on the map. This is the scale normally listed on a road map. Scale selection results in the amount of detail including roads, water bodies, and land use patterns.
- Geographic extent: Refers to the size of the study area. For example, a study can be regional scale or global scale. The extent of the study area and/or its subsets can affect the analysis results (e.g., different results might be obtained when looking at cancer incidence in one state or province versus nationwide).
- Spatial resolution: Refers to the grain, or smallest, unit that is distinguishable. Map data at different scales will allow for resolution of different objects. For example, a house site represented on a 1:24,000 scale map would not appear on a 1:100,000 scale map. In remotely sensed imagery, resolution is directly related to the pixel size, the area on the ground from which the radiances are integrated. Lower resolution pixel (1 km^2^) data may be less useful than higher resolution pixel Landsat data (30 m^2^) for some environmental health studies.
- Operational scale: Refers to the scale at which the process of interest occurs. For example, contaminant transport may occur at a small or large scale. Processes can be resolution dependent, that is, they can be detected at one scale but not another.

Homogeneity and heterogeneity of spatial data are affected by scale, and the scale chosen may affect the ability of the study to detect a relationship between the environmental exposure and the health outcome. This issue is similar to the modifiable areal unit problem, a term introduced by Openshaw and Taylor (1979) that has long been recognized as an issue in the analysis of aggregated data such as disease incidence rates and census enumeration (Fotheringham and Wong 1991; Holt et al. 1996). For example, studies of disease incidence reported at the county level require the environmental data to be aggregated to an exposure metric at the same resolution. Such aggregation may obscure intracounty variation in exposure (operational scale) and thus the relationship between the target contaminant and the disease.

Accuracy can be defined as how well the GIS data represent reality in terms of positional, attribute, and temporal accuracy. Positional accuracy relates to the agreement between data representation in the GIS and actual location of the data, or “ground truth.” Attribute accuracy is a measure of how well information linked to the data representation format is correct (e.g., is the line segment tagged with the correct street information?). Temporal accuracy concerns the appropriateness of using a particular snapshot or snapshots of time for a particular GIS-based analysis or modeling effort. For example, temporal accuracy would reflect how well using a single-year crop map would reflect proximity to pesticide use for exposure assessment of a particular disease outcome. Errors in GIS can be categorized as source errors or processing errors. Source errors relate to the accuracy of the data per se, that is, the differences between the data in the GIS and reality. For example, geocoding is often used to estimate the location of residences and pollutant sources; however, the positional error generated at this first step in the exposure assessment process is rarely evaluated. A study by Krieger et al. (2001) compared geocoding firms and found widely varying geocoding success rates as well as large differences in the accuracy of census tract assignment. The positional accuracy of geocoded addresses in epidemiology studies was evaluated in a breast cancer case–control study in western New York (Bonner et al. 2003) and in a non-Hodgkin lymphoma case–control study in Iowa (Ward et al., in press). The positional errors were comparable in the two studies; the majority of homes were geocoded to within 100 meters of their location determined by GPS. However, positional errors were greater for homes outside the large metropolitan areas (Bonner et al. 2003), and rural addresses in Iowa had a median positional error of around 200 meters (Ward et al. submitted).

Processing errors can be introduced into the database as a result of GIS-based analysis and modeling. For each layer of data combined in a GIS analysis, additional uncertainty in the analysis process will be introduced because of error propagation. Beyea and Hatch (1999) provide an in-depth discussion of uncertainty in GIS-based exposure modeling.

### Environmental Science

Environmental science is the systematic study of the complex of physical, chemical, and biotic factors that act upon on an organism or an ecologic community and ultimately determine its form and survival. It can include circumstances, objects, or conditions by which an organism or community is surrounded and the aggregate of social and cultural conditions that influence the life of an individual or community. Fundamental elements of environmental science relevant to GIS applications in exposure assessment include measurement data and predictive algorithms for fate and transport of chemical compounds in the environment.

Environmental science studies rely heavily on measurement data of the factors that influence life. Institutions in almost every country in the world, such as the U.S. Environmental Protection Agency (U.S. EPA), have been established with a primary mission of collecting and analyzing environmental samples to understand the impact of these factors on the health of the earth’s ecosystem. As a result, an abundance of measurement data concerning the chemical composition of air and water resources is available to environmental epidemiology studies. A basic principle in environmental sciences is that measurement data should be used within the bounds of the purpose for which the sample was collected. Often this purpose is to define regional or systematic trends in environmental quality at a scale and resolution that may not be adequate for epidemiologic studies, especially studies of individuals. For example, public water utilities operating in the United States with a service population > 10,000 are required by federal law to report levels of certain byproducts of the disinfection process to the U.S. EPA. Most utilities meet this requirement by taking four samples at different locations in their water distribution system every 3 months. Although this sampling design may be sufficient to indicate compliance with the law, it may not be sufficient to adequately encompass the spatial and temporal variability in exposure necessary to classify exposure to individuals using the water.

Environmental scientists often use computer-based simulation models to supplement measurement data in environmental studies. These models are generally composed of mathematic algorithms designed to predict interaction between, and effect of the complex factors on, an organism or ecologic community. The models can be stochastic (based on statistical probability) or deterministic (based on physical processes). In either case the models are dependent on measurement data for calibration of the predictive algorithms and validation of the predicted results. A fundamental rule in environmental modeling is not to transfer use of a model from one geographic region to another without validating it with measurement data from the new study area. Often such model transfer will require recalibration of the model as well. It is also a general rule in environmental modeling to reserve a statistically sufficient portion of available measurement data for model validation. Caution should also be employed in using a model at a spatial scale or temporal pattern for which it was not designed. A number of textbooks address environmental science and modeling (Clark 1996; Crawford-Brown 2001).

“Geophysical plausibility” is the term we have coined for use in application of environmental science to exposure assessment for epidemiology. In simplest terms this axiom would dictate that an association between a contaminant source and exposure to an organism or ecologic community cannot exist unless there is a plausible geophysical route of transport for the contaminant between the source and the receptor. For example, assume we are conducting a study of drinking water as the sole source of exposure to a specific contaminant and a disease outcome. If a landfill is leaching the contaminant into a groundwater resource (aquifer) in our study area, but our study population has always used another water supply source with no geophysical connectivity to the aquifer, it is implausible that the contaminant from the landfill is causing the adverse health outcome through a drinking water route of exposure. This axiom is particularly relevant in the use of GIS-based processing functions (e.g., kriging on measurement data) to develop exposure estimates in environmental epidemiology studies.

### Epidemiology

The fundamental guidelines for the design of an environmental epidemiology study are relevant whether or not GIS technology is being used for exposure assessment. A well-designed epidemiologic study takes into account potential confounding factors, including other exposures that may co-occur with the exposure of interest. The study should be designed to have adequate power to detect an association between the exposure and health outcome and to evaluate exposure–response relationships. For many environmental exposures the anticipated magnitude of the association with disease is likely to be modest, therefore a careful evaluation of the expected prevalence of exposure is critical to determining adequate study power.

A GIS can be used to evaluate the population potentially exposed and to determine if there is likely to be adequate variation in exposure across a study area. Wartenberg et al. (1993) used a GIS to develop an automated method for identifying populations living near high-voltage lines for the purpose of evaluating childhood leukemia and electromagnetic radiation. Another example is the use of a GIS to link disease registry information with public water supply monitoring and location data to determine potential study areas for evaluating the relation between disinfection byproducts exposure and adverse reproductive outcomes and cancer (Raucher et al. 2000).

The epidemiologic study should have the capability to evaluate the exposure in relation to an appropriate latency for the disease and to evaluate critical time windows of exposure. One limitation of a GIS is that mapped data often represent only one snapshot in time. However, several recent efforts have used GIS to reconstruct historical exposure to pesticides (Brody et al. 2002) and drinking water contaminants (Swartz et al. 2003) over a period of decades for a study of breast cancer on Cape Cod, Massachusetts. A study of fetal death in California (Bell et al. 2001) used an exposure metric based on agricultural pesticide use near the mother’s residence during specific time periods during the pregnancy.

Misclassification of exposure is of particular concern in environmental epidemiology studies because of the challenges in estimating exposure to environmental contaminants, which can occur across multiple locations and often at low levels. Exposure errors in time–series studies can occur as a continuum of measurement errors between classic-type errors and Berkson errors, as has been presented in detail by Zeger et al. (2000) regarding air pollution and health. Each type of error has different effects on the estimation of risk. Berkson error occurs when the exposure metric is at the population level, and individual exposures vary because of different activity patterns. An example of a population-level or aggregate exposure metric is the assignment of air pollutant levels from a stationary air monitor to the population living in the vicinity of the monitor. Berkson error does not lead to bias in the risk estimate although the variance of the risk estimate is increased (Zeger et al. 2000).

In a classic error model the exposure metric used in an epidemiologic study is measured with error and is an imperfect surrogate for the true exposure. If misclassification of exposure is nondifferential in terms of the health outcome, the effect is generally to bias risk estimates toward the null, thus potentially missing true associations (Copeland et al. 1977; Flegal et al. 1986). To evaluate the degree of misclassification that may occur in an epidemiologic study, it is important to consider the sensitivity and specificity of the exposure metric employed. Sensitivity is the ability of an exposure metric to correctly classify as exposed those who are truly exposed. Specificity is the ability of the metric to correctly classify as unexposed those who are unexposed. Most epidemiologists do not formally assess the validity of their exposure metric before a study is launched; however, small reductions in sensitivity and/or specificity of the exposure metric can have substantial effects on the estimates of risk. When the true prevalence of exposure is low (e.g., less than 10%) small reductions in specificity cause substantial reductions in the risk estimates, whereas reductions in sensitivity have smaller effects. When the exposure is common in the study population, the sensitivity of the exposure metric becomes more important (Stewart and Correa-Villasenor 1991).

A common metric used in studies employing GIS is the proximity between a pollutant source and a residence. Simple proximity metrics are likely to overestimate the population truly exposed (high sensitivity but low specificity). If those truly exposed represent only a small percent of the study population, there will be substantial attenuation of the risk estimate if a true risk exists. Rull and Ritz (2003) compared several methods of classifying a study population in California on the basis of agricultural pesticide use reported by the California Pesticide Use Reporting (CPUR) database (http://www.cdpr.ca.gov/). The prevalence of exposure differed substantially depending on the metric used. They assumed that a metric that accounted for the location of crop fields more accurately represented true exposures and this metric resulted in lower exposure prevalence compared with a metric based on the CPUR database alone. In a simulation study they demonstrated that the reduced specificity of the CPUR metric resulted in substantial attenuation of risk estimates.

## Using GIS to Define the Study Population in an Epidemiologic Study

When epidemiologists select a study population, they are, by default, defining a system boundary for the exposure assessment process. This system boundary is an important element of source-receptor modeling approaches that may be used in the exposure assessment process. Location data for the study population are typically a set of geopolitical units (census enumeration unit boundaries) or the actual residences of the study population. Both of these data types can be represented using functions common to most GIS software. Usually, the subjects are identified from health registries or other records that identify individual cases or disease rates in a geographic area. Examples include cancer registry data, hospital records of a particular disease outcome, or death certificate data. Many of these data are now stored digitally, and an increasing percentage are also georeferenced so that transfer to a GIS database is possible. Controls are identified and located by the epidemiologist, often by frequency-matching characteristics of each case subject that are relevant to disease etiology, including age and sex. Controls are usually selected from the same general geographic region, which should represent the base population from which the cases arise.

### Example: Classification of Populations near Landfill Sites (Elliott et al. 2001)

**Figure 3:**
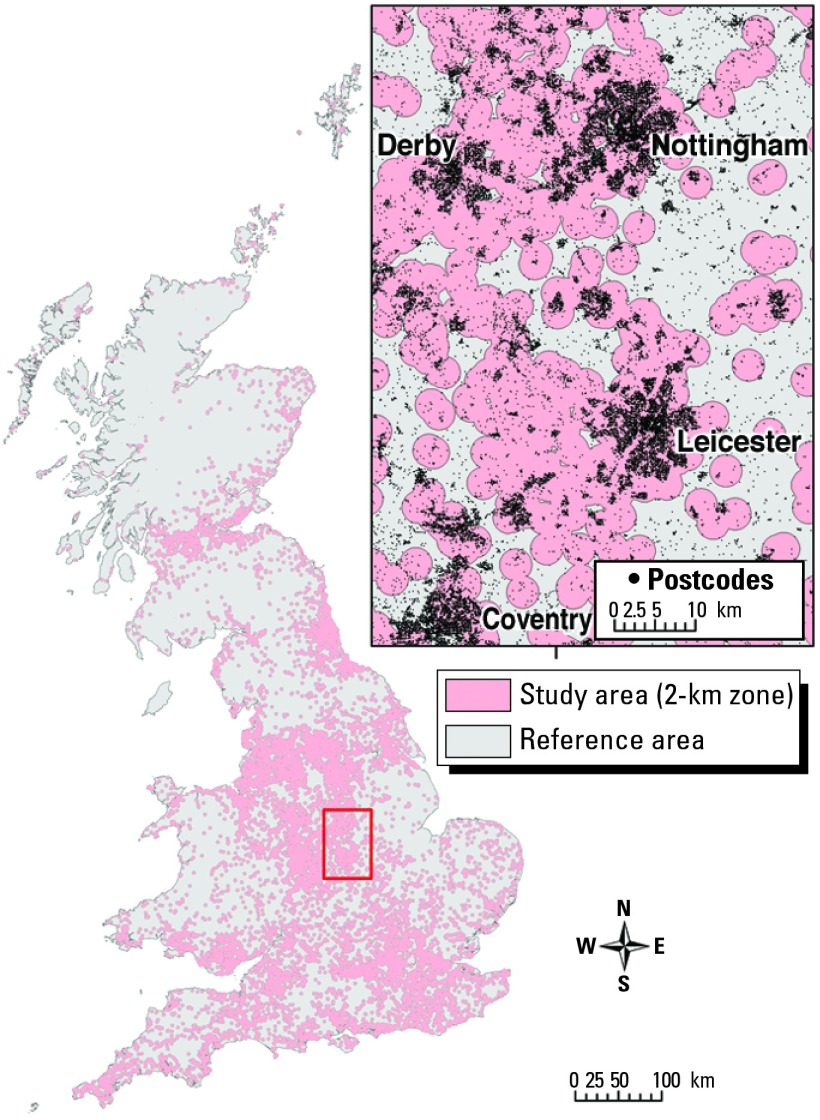
Distribution of landfill sites in the Great Britain, buffered to 2 km, with an inset showing details of the buffer zones in pink (SAHSU 2001). The high density of sites in many areas results in considerable overlap of the buffer zones used to define exposures, and thus means that many areas are classified as exposed from a number of different landfill sites (see inset).

Public concern has been raised that living near a landfill site may be hazardous to health. In particular, several U.S. and U.K. studies have shown excess risk of birth anomalies in populations living near landfill sites (Dolk et al. 1998; Fielder et al. 2000; Vrijheid 2000). To investigate potential risk of adverse birth outcomes associated with landfill sites in Great Britain, investigators had access to an extensive data set of current and previously opened landfill sites provided by the environmental protection agencies in Great Britain. Data were incorporated in a GIS, resulting in a database containing 19,196 landfill sites in England, Wales, and Scotland. Detailed data on boundaries were unavailable for most sites, and therefore point locations had to be used. Site centroids were given for a majority of sites. The location of the site gateway at the time of reporting was used for the remainder. Geocoded data were supplied for landfill site locations but were of low accuracy (often rounded to 1,000 m), and area data were inadequate for most sites. Landfill site areas also changed considerably over time. Postcodes, which were used to define the location of cases and births, only approximated the place of residence. When researchers tried to intersect location of landfill(s) and residences of study subjects, they found that landfill sites are often highly clustered, so that individual postcodes may lie close to as many as 30 or more sites. Given that study subjects may be exposed to several landfill sites, distance from the nearest landfill site was not regarded as a meaningful proxy for exposure. As a compromise between the need for spatial precision and the limited accuracy of the data, a 2-km zone was constructed around each site (Figure 3), giving a resolution similar to or higher than that of previous studies (Dolk et al. 1998; Fielder et al. 2000) and at the likely limit of dispersion for landfill emissions (WHO 2001). The reference population comprised people living more than 2 km from all known landfill sites during the study period. Availability of landfills and health outcome data were restricted to the study period from 1983 to 1998.

Because health data were available only to 1998 and because of concerns about the quality of the early landfill data, 9,631 sites that closed before 1982 or opened after 1997 were excluded (allowing a 1-year lag period for the birth outcomes), as were landfill sites for which there were inadequate data. The remaining 9,565 sites included 774 sites for special (hazardous) waste, 7,803 for nonspecial waste, and 988 handling unknown types.

The study was the largest performed on possible associations between residence near landfill sites and adverse birth outcomes. A GIS-based approach was necessary because of the large number of landfill sites included in the study; individual investigations of several thousand landfill sites would have been practically difficult and prohibitively expensive. The most striking finding was that approximately 80% of the British population live within 2 km of a landfill site. This also imposed unique challenges for the epidemiologic study design, given that 80% of the study population was potentially exposed and only 20% could be used as a reference. In most environmental epidemiology studies, the situation is the opposite in that the prevalence of those potentially exposed is much lower. This high prevalence of potential exposure had implications for the statistical analysis, as the usual reference rates after stratification by known confounders would not be estimated with the negligible error normally associated with such studies. Despite this, the reference area included over 2 million births over the study period. To guard against overinterpretation, 99% (rather than the more commonly used 95%) confidence intervals around the relative risk (RR) estimates were computed.

The authors were aware of the relative inaccuracy of postcodes (used to define the location of cases and births), as these give only an approximation of place of residence, accurate to 10–100 m in urban areas but > 1 km in some rural areas. Furthermore, it is well known that postcodes are afflicted with several other problems: they may change over time, be terminated, or even recycled. However, such problems affect only a small minority (approximately 1%) of U.K. post-codes. Thus, given the size of the study (national rather than local), this is not a major problem. For further details the reader is referred to the original article (Elliott et al. 2001).

## Using GIS to Identify Source and Potential Routes of Exposure in an Epidemiologic Study

The exposure or agent of interest in an environmental epidemiology study may be a chemical (a single compound or, rarely, a mixture) or physical agents (particulates, radiation, noise). Once the agent is identified, a GIS can be instrumental in identifying sources and potential routes of exposure. Source identification is a function of the occurrence of the target agent in a specified environmental medium (air, water, food, dust, etc.). Identifying the sources enables assessment of the likelihood of exposure across the study population and provides data on the route of exposure information necessary for calculating personal exposure.

### Example: Neurobehavioral Effects of Exposure to Trichloroethylene through a Municipal Water Supply (Reif et al. 2003)

**Figure 4:**
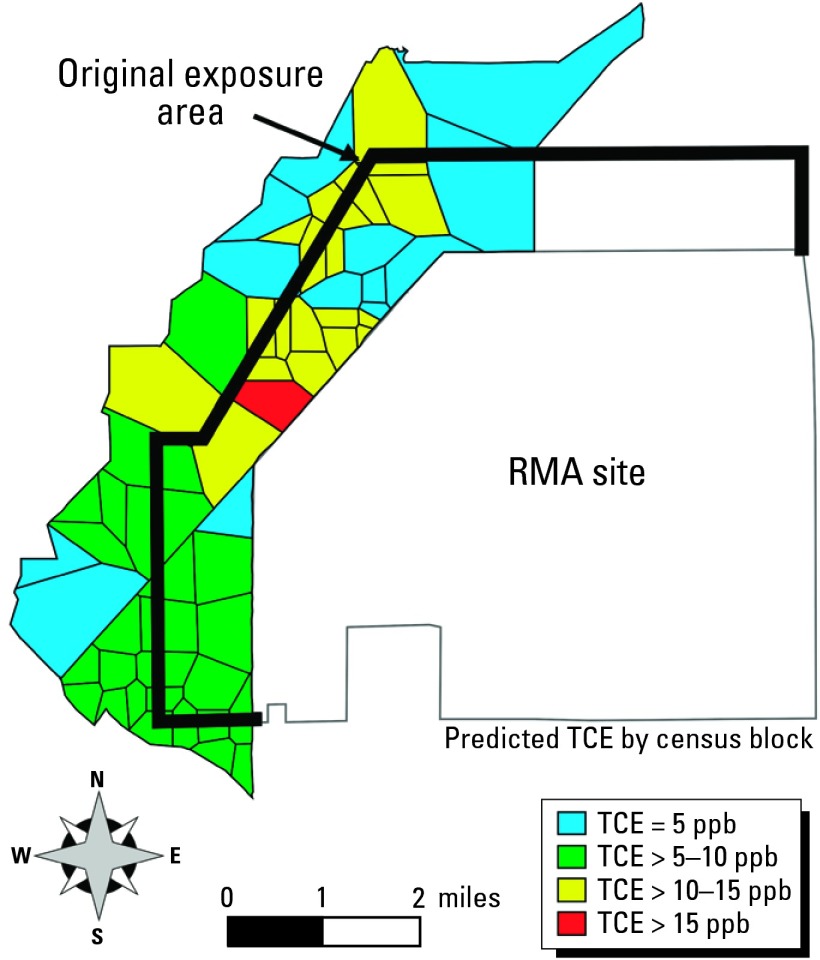
Exposure zone in original RMA study (ATSDR 1996) and refined resolution of predicted exposure to TCE by census block as reported by Reif et al. (2003).

The basis for this study was initially a cross-sectional study of exposure to a number of chemicals with documented release in a community adjacent to a Superfund waste site, the Rocky Mountain Arsenal (RMA) near Denver, Colorado, USA. Study participants were randomly selected from an area within 1.61 km (1 mile) that abutted to the north, northwest, and west boundaries of the site, where fugitive chemicals had been detected in ground and surface waters, sediments, and soils (Figure 4). A total of 585 persons who had lived at their current residence for at least 2 years were eligible for the study; 472 participated. Results of the initial study warranted a second study, conducted in 1991, during which the researchers interviewed and conducted neurobehavioral testing of 204 adults originally identified by the first study (ATSDR 1996). Results of the 1991 study showed a trend toward an increased prevalence of neurologic disorders and adverse reproductive outcomes, particularly in the area north/northwest of the RMA, compared with communities at a greater distance from RMA, presumed to be unexposed to the site. However, the researchers again relied on proximity to the RMA as a surrogate for exposure, and there was evidence that this may have resulted in nondifferential misclassification of exposure, which tends to drive the effect estimate or relative risk toward the null value (Copeland et al. 1977). The researchers initiated a revised exposure assessment using a GIS-based analysis of fate and transport of chemicals in the groundwater regimen hydraulically downgradient from the RMA site. The researchers selected trichloroethylene (TCE) as the marker contaminant for the exposure assessment because of its neurotoxi-cologic properties, and because it had been detected in water supply wells in the study area. The researchers constructed an operable MODFLOW (U.S. Geological Survey, Reston, Virginia, USA) simulation model that accurately reflected hydraulic characteristics of groundwater regime in the study area and used a GIS to develop input variables to the model, including source location of TCE on the RMA site. However, the researchers could not validate TCE levels measured in water wells used by the local water district (LWD), where 90% of the study population resided. The researchers expanded the geographic extent of their study area, and determined that the source of TCE in the groundwater was from multiple hazardous waste sites, including some located outside the original study area. Once the primary source was properly identified, the researchers confirmed the measurement results of TCE in the LWD supply wells by the groundwater model. TCE levels in the wells were then used as input to a hydraulic and water quality simulation model, EPANET (Rossman 1994), to predict TCE levels in the distribution system of the LWD. The researchers used GIS to geocode the study population, develop input data for the simulation model, and assign individual exposure to TCE by linking results of the model to the census block group of residence (Figure 4). The study with the refined exposure assessment found a stronger association of risk for neurobehavioral disorders in the study population than was found in the 1991 study, in which exposure was based primarily on proximity to a source of chemical contamination, including TCE. The study demonstrates that GIS-based technology can be used to refine exposure for epidemiologic investigations, improving sensitivity and specificity beyond a simple proximity metric. It also demonstrates the effect that selection of operational scale can have on exposure assessment in an epidemiology study. The operation of the water distribution system could not be discerned when proximate census blocks were used as a surrogate for exposure.

## Using GIS to Estimate Environmental Levels of Target Contaminants in an Epidemiologic Study

Exposure is a function of the concentration of target contaminant in the environment of the study population. The optimal method for quantifying levels of the target agent is the measurement of the environmental media associated with each potential route of exposure during the critical time period for exposure. However, rarely is there an opportunity to make such measurements. Alternatively, predicted environmental levels of the target agent can be estimated using source-receptor modeling. Computer-based models designed to predict levels of contaminants due to point sources (smokestack) or nonpoint sources (drift from aerial spraying of pesticides) are available. Often these predictions are used as a surrogate for exposure in the epidemiologic analysis. In either case, validation of the estimates is important to understand the results of the epidemiologic study. Validation is often overlooked in the exposure assessment process. It is also important that the environment depicted in the modeling period be representative of the environment during the exposure period necessary for the epidemiologic study. Generally, the degree to which validation can be accomplished is a function of measurement data available for the time period of interest. Most source-receptor models require some measurements for constructing (calibrating) the predictive algorithms.

### Example: The Lung Cancer in Stockholm Study (Bellander et al. 2001; Nyberg et al. 2000)

A population-based case–control study, the Lung Cancer in Stockholm Study (LUCAS), was designed to investigate whether urban air pollution increases lung cancer risk. Previous studies had commonly used crude surrogates for individual exposure, limiting the power of detecting any risk associated with air pollution. The LUCAS study used advanced modeling techniques to assess individual exposure for relevant time periods (several decades before diagnosis). Detailed emission data, dispersion models, and GIS were used to assess historical exposure to several components of ambient air pollution. The study base consisted of all men 40–75 years of age who lived in Stockholm County at any time between 1985 and 1990 and who had lived in the county since 1950, with a maximum of 5 years of residence outside the county. A total of 1,042 lung cancer cases diagnosed between 1985 and 1990 were included, as well as 2,364 controls. Information on residence from 1955 to the end of follow-up for each individual, 1990–1995, was collected using a questionnaire. Nitrogen oxides (NO_x_ and NO_2_) and sulfur dioxide (SO_2_) were chosen as indicators of air pollution from road traffic and residential heating, respectively.

**Figure 5:**
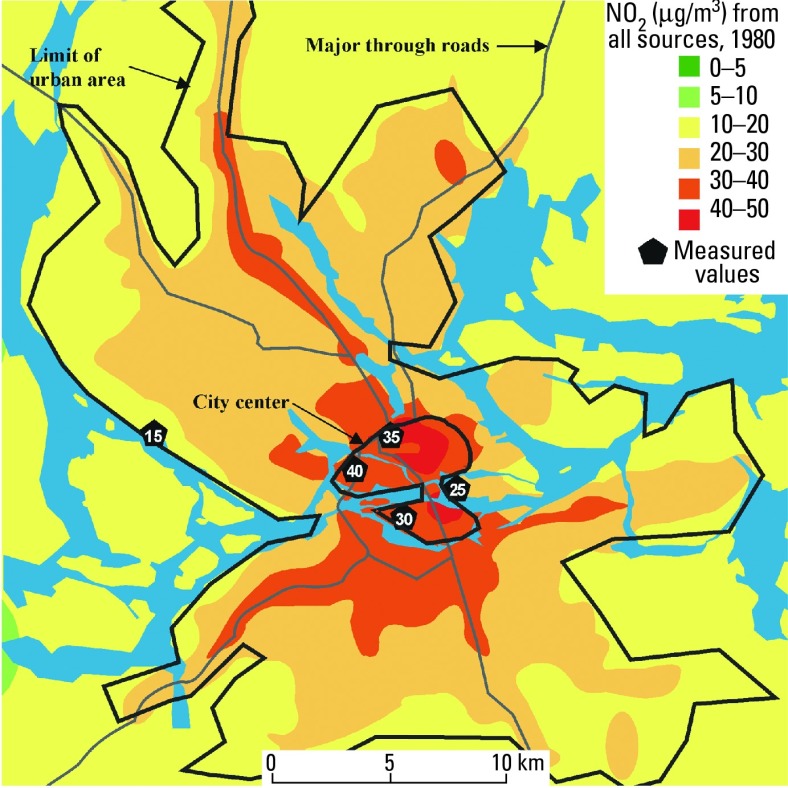
Modeled ambient air concentrations of NO_2_ emissions from all sources (1980 data) using reconstructed emission data for this index pollutant together with dispersion modeling (Bellander et al. 2001).

Ambient air concentrations of NO_x_ , NO_2_, and SO_2_ were assessed throughout the study area for three points in time (1960, 1970, and 1980) using reconstructed emission data for these index pollutants together with dispersion modeling (Figure 5). The modeled NO_2_ estimates for 1980 were validated with available measurement data. Linear intra- and extrapolation were used to obtain annual estimates for the remainder of the exposure period (1955–1990). Individual addresses were geocoded with an estimated error of < 100 m for 90% of the addresses. Annual air pollution estimates were then linked to residence coordinates, yielding cumulative residential exposure indices for each individual. There was a wide range of individual long-term average exposure, with an 11-fold interindividual difference in NO_2_ and an 18-fold difference in SO_2_

The detailed individual exposure assessment made it possible to assess relative risk potentially associated with road traffic. Average traffic-related NO_2_ exposure over 30 years was associated with a relative risk of 1.4 and a 95% confidence interval 1.0, 2.0 for the top decile of exposure, adjusted for tobacco smoking, socioeconomic status (SES), residential radon, and occupational exposures, and taking into consideration a latency period of 20 years (Nyberg et al. 2000). The signifi-cance of these results was recognized in an accompanying editorial as being the first study that had used this advanced exposure assessment, making the detailed analysis possible (Rothman and Cann 2000).

The results indicate that GIS can be useful for exposure assessment in environmental epidemiology studies, provided that detailed geographically related exposure data are available for relevant time periods.

## Using GIS to Estimate Personal Exposure in an Epidemiologic Study

A key issue in exposure assessment is how well an exposure metric estimates exposure to the individual. Exposure has been defined as “the contact of a chemical, physical, or biological agent with the outer boundary of an organism” (Berglund et al. 2002). Exposure is a function of concentration and time: “An event that occurs when there is contact at a boundary between a human and the environment with a contaminant of a specific concentration for an interval of time” (NRC 1991). Thus, in the context of exposure assessment for an epidemiologic study, it is important to distinguish between environmental concentration, exposure concentration, and dose. The environmental concentration of an agent refers to its presence in a particular carrier medium [for example, polycyclic aromatic hydrocarbons (PAH) in ambient air], expressed in quantitative terms (for example, micrograms per cubic meter). Similarly, the exposure concentration of an agent refers to its presence in its carrier medium at the point of contact (for example, PAH in breathing zone air) expressed in quantitative terms (for example, micrograms per cubic meter). Finally, the dose refers to the amount of a pollutant that actually enters the human body, i.e., is taken up through absorption barriers. A number of variables can influence the exposure and dose. These include physiologic factors such as age, sex, physical condition, disease, and genetics, as well as exposure factors related to human behavior and activities (e.g., the amount of time spent commuting to work each day), and contact rates (e.g., the amount of drinking water ingested per day). In epidemiologic studies, environmental concentration will often be used as a surrogate for both exposure concentration and dose.

We could not find an example of the use of GIS to estimate personal exposure for an epidemiologic study. In our review of the literature, questionnaire data were generally used as a surrogate for deriving personal exposure. Only recently have researchers started using GIS to study activity patterns in a study population, which conceivably could be linked to environmental data for exposure assessment. Phillips et al. (2001) reported on a test of GPS data recorders as a means of validating time-location data recorded in study diaries of a subset of participants enrolled in the Oklahoma Urban Air Toxics Study. Elgethun et al. (2003) describe the development and testing of a data-logging GPS unit designed to be integrating into clothing. Both studies concluded that GPS units could be useful in developing time–location information for use in exposure assessment. GPS is a satellite-based technology composed of a system of satellites encircling earth and emitting a radio frequency detectable by GPS receivers. GPS receivers are designed to use this information and calculate coordinates of the receiver location. Precision of these coordinates can vary based on receiver design and signal quality. Phillips et al. (2001) reported precision of about 10 m for most readings, whereas Elgethun et al. (2003) reported mean root mean square error of 3.2 m outdoors and 5.8 m indoor in positional accuracy for two GPS units tested. This level of precision should be sufficient for most studies attempting to link location of a participant with a particular environmental setting where contaminant monitoring or modeling data are available for linkage using a GIS. A major advantage of the technology, as reported by Phillips et al. (2001), was not only that its use confirmed all reported trips over a 12- to 23-hr monitoring period, but it provided time–location data on travel events not recorded in the participant diary.

Both Phillips et al. (2001) and Elgethun et al. (2003) reported limitations of the technology as a sole source of space–time data for an exposure assessment study. Both studies found the reception of the satellite signals to be adversely impacted by shielding from buildings of certain materials (concrete, steel), electrical power stations, and to some extent vehicle body panels. Signal blockage continues to be an issue with GPS today. Phillips et al. (2001) also reported extensive failure including battery failure, data-logging failure, and data storage limitations, which resulted in capturing only about 30% of the total monitoring time attempted in 25 trials. Elgethun et al. (2003) reported reception efficiencies of 79% outdoors, 20% in homes, 12% in vehicles, and 6–9% in schools and businesses. These findings indicate that although GIS using GPS technology hold promise in terms of integrating study population activity data with measured or predicted levels of environmental contaminants in the exposure assessment process, their use is still very much in the developmental research stage for use in epidemiology studies.

## Discussion

Our findings indicate that GIS can greatly enhance epidemiologic research in terms of definition of source and routes of potential exposure and estimation of environmental levels of target contaminants in the exposure assessment process. We found over 15 studies published since 1998 that describe the successful use of GIS for one or more of these purposes. Across all of these studies, there was consensus that the use of GIS was instrumental in achieving optimal exposure assessment. In our example studies, GIS improved resolution of the source of potential exposure (Elliott et al. 2001; Reif et al. 2003), identified the most likely route of exposure (Reif et al. 2003), and estimated levels of target contaminants for use in estimating exposure to the study population (Nyberg et al. 2000; Reif et al. 2003). Our examples of environmental epidemiology studies using GIS also emphasize the importance of interdisciplinary study teams.

GIS have been used to evaluate environmental justice issues, usually by linking information about potential sources of environmental pollutants to census information on sociodemographic characteristics of a population (Perlin et al. 2001; Waller et al. 1999). However, only recently have GIS been used in the design of environmental epidemiology studies. Each example in our article demonstrates that GIS can (and perhaps should) be used in the early planning stages of an environmental epidemiology study to help locate a potential study population with a wide range of exposure. The statistical power of an epidemiologic study and the precision of the risk estimates are optimized when the study population includes adequate numbers of those with both high and low exposures. An example of how GIS have been used to identify a study population with a range of exposures is a feasibility study of childhood leukemia and electromagnetic radiation from power transmission lines in New Jersey (Wartenberg et al. 1993). A GIS was used to identify the population living close to transmission lines and a comparison population farther away. Demographic information was evaluated for both the exposed and unexposed populations to determine potential confounding factors. Other examples include the use of GIS for surveillance and study of lead poisoning from residential exposures (Roberts et al. 2003; Wartenberg 1992).

The increasing availability of environmental databases in a geographic format (Paulu et al. 1995), including the location of industrial sites and releases (Toxic Release Inventory Program 2004), should make it feasible to incorporate these potential exposure data into epidemiologic studies. For example, in a recently started cross-sectional study on potential adverse health effects (primarily hypertension) of airport-related noise exposure, study populations are being selected using modeled noise contours around the participating airports (European Commission 2003). Such models are particularly applicabile in the selection of study populations exposed to different levels of the pollutants under study, using a cross-sectional or cohort study approach. A case–control design, in which cases are selected from, for example, hospital data or cancer registries, will usually have a predefined area (hospital catchment or cancer registry area); thus, preexisting exposure information may be less relevant in the study population selection. However, exposure information can be used to delimit the study area within the bounds of the catchment area or disease registry. For example, AWWA (2000) demonstrated the feasibility of linking environmental monitoring data with birth and cancer registry data to identify optimal geographic locations for epidemiologic studies of by-products of chlorination in public water supplies in the United States. GIS also have potential uses in the selection of controls for an epidemiologic study, as they are usually randomly selected from the same geographic area as the cases. As frequency matching (on age and sex) is commonly applied for study efficiency reasons, GIS could also be used for further frequency matching on SES, where areas are classified according to a georeferenced SES index.

There are, of course, a number of caveats regarding use of GIS for exposure assessment in environmental epidemiologic studies. We reviewed fundamental principles of three scientific disciplines critical to such applications: geospatial science, environmental science, and epidemiology. Axiomatic themes from each of these scientific disciplines should be adhered to in any case, but they are particularly relevant when using a GIS. These themes include accuracy and validity of data (raw and calculated), appropriate selection of mathematic formulas and models, and scientific plausibility. The application of these axiomatic themes can be very different across the scientific disciplines, which reinforces the need for multidisciplinary teams in conducting environmental epidemiology studies. For example, researchers in each of the disciplines are trained in determining the accuracy and precision of measurement data. However, only the geospatial scientist or geographer is generally trained to rectify geographic data so that two or more GIS-based data layers such as health outcome and environmental data can be merged and the resulting data layer used to determine the association more accurately. Similarly, only the epidemiologist is likely to be trained to search for and identify other data layers that, if omitted from the test of association, could confound the results.

Use of measured environmental data and mathematic algorithms for estimating contaminant levels in exposure assessment is another area requiring specialized expertise in most cases. Since the advent of the computer age, packaged software has become more and more prevalent for such applications, but the old modeler adage “garbage in, garbage out” is perpetual truth. Even with the color maps produced using a GIS, “mapped garbage” is still “garbage.” In this article we propose several fundamental principles of environmental science and modeling that should be adhered to when using GIS in exposure assessment for epidemiology studies. Perhaps the most important of these principals can be captured by the term “validation.” In each of our example studies, environmental data were used to develop an exposure metric for use in epidemiology. The data used were collected for other purposes, commonly for administrative or regulatory use. These studies demonstrate the range of measurement data quality and degree of validation that may be possible from relatively low (Elliott et al. 2001) to high (Nyberg et al. 2000). They also demonstrate the likely consequences across this range in terms of risk estimates in an epidemiology study. In Elliott et al. (2001), a database on landfill sites was obtained from the environmental protection agencies, which collected the data from site operators in the licensing process. Thus, data that would have been useful for exposure assessment were not readily available (e.g., volumes and types of waste actually received at the landfill sites, measurement data for specific chemicals being released into the environment, or the extent of contamination). Instead, the likely limit of dispersion for landfill emissions (2 km) was estimated based on published information and used as an exposure boundary around each site, degree of hazard for exposure was derived from the type of license held by the operator, and the epidemiologic analysis assumed a common relative risk for all landfill sites. The researchers did not validate these exposure metrics. It is likely that sites licensed to carry special (hazardous) waste did not necessarily do so, and that sites licensed to carry nonspecial waste actually did carry some hazardous waste as well. The resulting exposure misclassification was most likely nondifferential, which could result in a bias risk estimate toward the null (Copeland et al. 1977). The findings of the study, small excess risks for some birth outcomes after exposure to landfills, seem to verify this conclusion.

The study reported by Reif et al. (2003) concerning TCE and neurobehavioral demonstrated that improvement in exposure assessment techniques “refined exposure . . . with adequate specificity to reveal adverse effects [of TCE] in the nervous system.” In that study, the researchers refined exposure assessment by replacing a proximity metric such as the one used in Elliott et al. (2001) with exposure predictions based on validated environmental measurements (TCE levels in groundwater at source wells for a municipal water system) and validated transport modeling (water pressure and volume in the municipal water system) during the exposure period for the study. However, data were not available to validate predicted TCE levels at study participants’ residences.

In the final example study that we reviewed, Bellander et al. (2001) had sufficient source emission and environmental measurement data to calibrate and validate predicted levels of NO_2_ in the environment of Stockholm, Sweden, for at least a portion of the exposure period in an epidemiologic study of lung cancer (1955–1990). They also validated their predicted location of residence in Stockholm for each participant in the study by cross-checking results using external geocoding service companies. The resolution and precision of this exposure assessment process resulted in the capability to detect a wide range of individual long-term average exposure and to detect risk of lung cancer to average traffic level exposure to NO_2_ within a 95% confidence limit. The procedures and results of these studies clearly indicate the need for expertise in environmental science and related disciplines in epidemiologic studies involving pollutant emissions.

## Conclusion

In summary, we have reviewed the recent literature on the use of GIS in exposure assessment for environmental epidemiology and described principles and applications of three core scientific disciplines needed, in our opinion, to successfully implement such studies: geospatial science, environmental science, and epidemiology. This by no means preempts the need for other scientific disciplines in the execution of such studies. In particular, statistics is a core science that would benefit every study, and other disciplines should be included based on the focus and objective of the study. Based on our findings, we offer the following conclusions:

- The use of GIS in exposure assessment for environmental epidemiology studies is not only feasible but can enhance the understanding of the association between contaminants in our environment and disease.
- A good environmental epidemiology study design should aim to maximize exposure contrasts and thus study population selection should be based on an a priori conception of the geographic distribution of exposures in the study area whenever possible (even if crude). For this purpose, GIS-based exposure mapping can be useful, given that geo-referenced data are available at a relevant scale.
- It is preferable in an environmental epidemiology study to estimate and validate levels of the agent (contaminant) of interest in the environment of the study population. These levels are the basis for estimating personal exposure and dose and for classifying exposure across a study population. GIS and related technology (source/receptor model; environmental simulation models) can improve accuracy in identifying source and route of potential exposure in a study area and in estimating levels of target contaminants.
- When environmental levels of the agent (contaminant) of interest in the environment of the study population cannot be measured or accurately predicted, GIS provide the optimal technology for using proximity to contaminant source in an environmental epidemiology study. It is well established as a viable tool in ecologic study design.
- GIS and related technologies such as the GPS are useful for providing precise locations of study participant residences and other stationary data. Research is needed on how to integrate this use of the technology with epidemiologic questionnaire and environmental data for exposure assessment.
- Environmental epidemiology studies require interdisciplinary expertise and adherence to the fundamental principles of geospatial science, environmental science, and epidemiology.

## Figures and Tables

**Figure 1:**
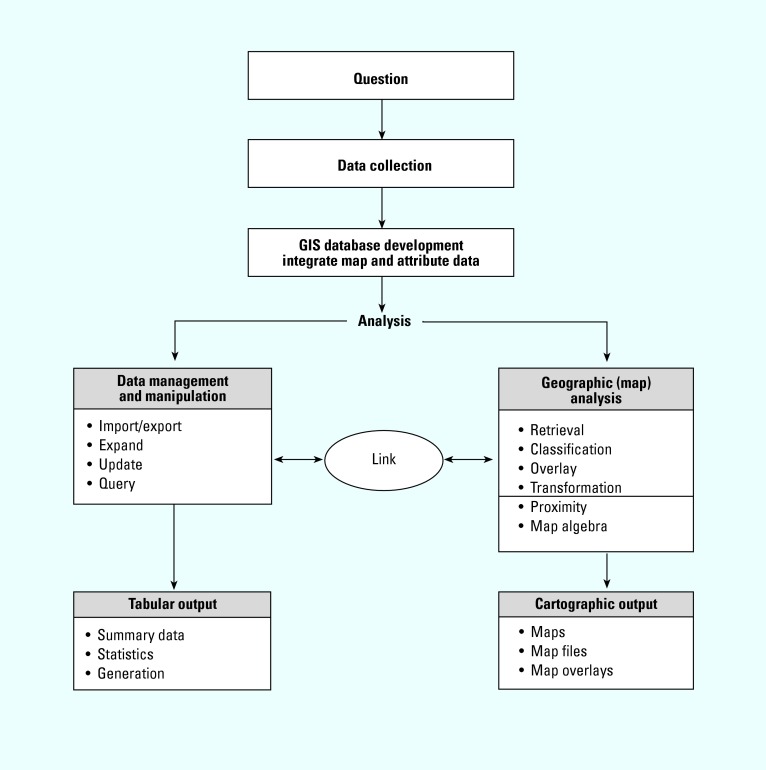
Structure and functionality of a GIS.

**Figure 2:**
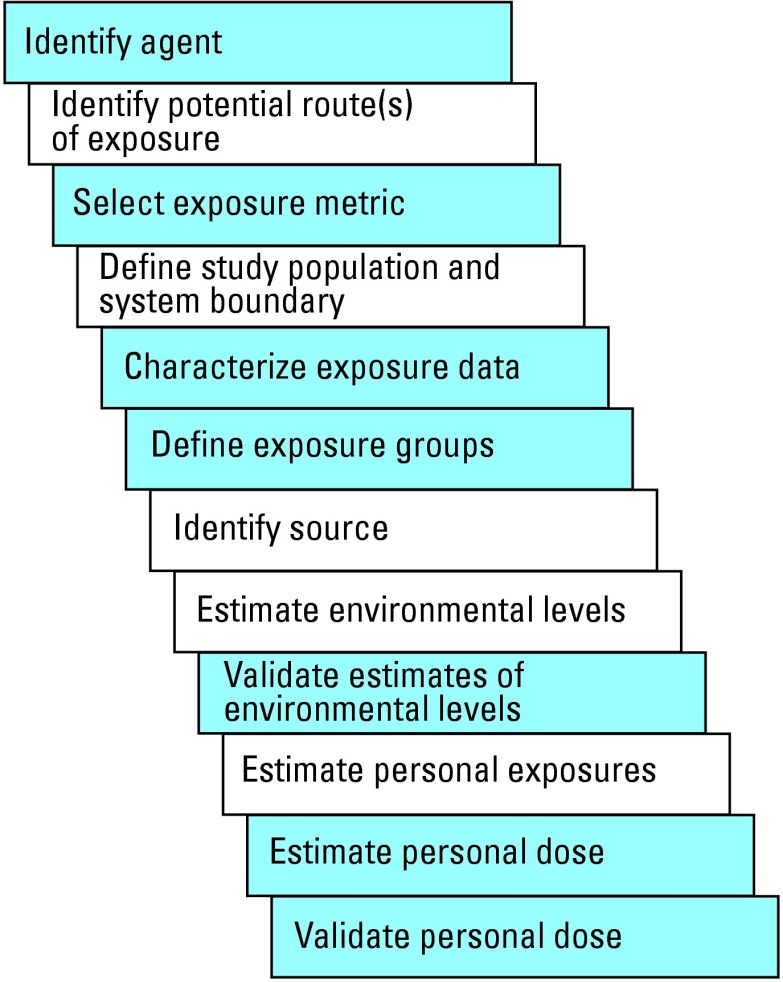
Exposure assessment process. Steps for which use of GIS is discussed in this article are highlighted in blue.
